# Efficacy of Continuous Epidural Analgesia versus Total Intravenous Analgesia on Postoperative Pain Control in Endovascular Abdominal Aortic Aneurysm Repair: A Retrospective Case-Control Study

**DOI:** 10.1155/2014/205164

**Published:** 2014-04-07

**Authors:** Ahmet Şen, Başar Erdivanlı, Abdullah Özdemir, Hızır Kazdal, Ersagun Tuğcugil

**Affiliations:** Department of Anesthesiology and Reanimation, Faculty of Medicine, Recep Tayyip Erdogan University, 53100 Rize, Turkey

## Abstract

We reviewed our experience to compare the effectiveness of epidural analgesia and total intravenous analgesia on postoperative pain control in patients undergoing endovascular abdominal aortic aneurysm repair. Records of 32 patients during a 2-year period were retrospectively investigated. TIVA group (*n* = 18) received total intravenous anesthesia, and EA group (*n* = 14) received epidural anesthesia and sedation. Pain assessment was performed on all patients on a daily basis during rest and activity on postoperative days until discharge from ward using the numeric rating scale. Data for demographic variables, required anesthetic level, perioperative hemodynamic variables, postoperative pain, and morbidities were recorded. There were no relevant differences concerning hospital stay (TIVA group: 14.1 ± 7.0, EA group: 13.5 ± 7.1), perioperative blood pressure variability (TIVA group: 15.6 ± 18.1, EA group: 14.8 ± 11.5), and perioperative hemodynamic complication rate (TIVA group: 17%, EA group: 14%). Postoperative pain scores differed significantly (TIVA group: 5.4 ± 0.9, EA group: 1.8 ± 0.8, *P* < 0.001). Epidural anesthesia and postoperative epidural analgesia better reduce postoperative pain better compared with general anesthesia and systemic analgesia, with similar effects on hemodynamic status.

## 1. Introduction

Abdominal aortic aneurysm is common in men older than 65 years of age. Most of these patients have a history of chronic tobacco smoking, chronic obstructive pulmonary disease, hypertension, and hyperlipidaemia [1]. Smoking and pulmonary disease may cause postoperative respiratory failure following general anesthesia due to increased atelectasis [2]. Inadequate postoperative analgesia may also promote atelectasis formation due to the patient's inability to cough. Conversely, use of rescue analgesics may cause hemodynamic fluctuations in such hypertensive patients and precipitate hemorrhagic or ischemic complications. Epidural analgesia is shown to improve hemodynamic stability and postoperative analgesia, alone [3] or combined with general anesthesia [4] in major abdominal surgery. We reviewed endovascular abdominal aortic aneurysm repair (EVAR) cases over a 2-year period and compared epidural anesthesia with general anesthesia in terms of perioperative hemodynamic stability and postoperative analgesia.

## 2. Materials and Methods

Following approval by the local ethics committee, we retrospectively investigated records of patients who had undergone EVAR surgery during the period from October, 2011, to October, 2013, in our institution. Patients, who received spinal anesthesia and combined regional and general anesthesia were excluded. One patient, who was operated with general anesthesia for 490 minutes due to accidental vascular tear, massive blood loss, and cardiac arrest during operation, was also excluded.

### 2.1. Data Collection

Two anesthesiologists reviewed the charts of all eligible patients. The following data were obtained: (1) demographic variables and medical history (age, gender, American Society of Anesthesiologists (ASA) risk score, and comorbidities) and cardiovascular events such as arrhythmia and treatments, baseline resting blood pressure, and heart rate were obtained from ward charts; (2) anesthesia method, drug doses, recordings of continuous electrocardiographic, pulse oximetry, urine output, central venous pressure (via internal jugular or subclavian vein catheterization), invasive blood pressure monitoring (via radial artery cannulation), intraoperative fluid therapy, urine output, blood loss and transfusion, hemodynamic events, and treatments were obtained from anesthesia charts; (3) blood pressure, heart rate, fluid therapy, urine output, postoperative pain (pain assessment was performed on all patients on a daily basis during rest and activity until discharge from the intensive care unit (ICU) using the numeric rating scale (as described in Table 1) [5]), analgesic requirement, and morbidities were obtained from the ICU chart.

### 2.2. Statistics

Normality of distributions was tested with Shapiro-Wilk test. Parametric data were presented as mean ± standard deviation (SD), nonparametric data were presented as median ± SD, and categoric data were presented as number (%). Parametric data (age, hospital stay, nutrition and fluid intake, and duration of postoperative active warming) were analyzed with student *t*-test, nonparametric data (operation duration, blood pressure values, heart rate values, intravenous fluids, urine output, occurrence of hemodynamic complications, and pain scores) were analyzed with Mann-Whitney *U* test, and categorical data (gender and ASA scores) were analyzed with chi-square test. The relationship between continuous variables such as intravenous fluids or urine output and duration of operation was analysed with analysis of variance (ANOVA); a *P* < 0.05 was considered statistically significant.

## 3. Results

Data from a total of 31 (27 males and 4 females) patients were analyzed. We found that 17 patients (TIVA group) received total intravenous anesthesia (TIVA) and 14 patients (EA group) received epidural anesthesia. Patients' demographic data were summarized in Table 2. There were no statistically significant differences between patients receiving either form of anesthesia in terms of gender, age, and comorbidities. Hospital stay did not differ significantly between TIVA group (14.1 ± 7.0 days) and EA group (13.5 ± 7.1 days).

### 3.1. Anesthesia Method

It is understood that, following an intravenous (iv) bolus of 3 mg midazolam, epidural catheterization was performed at sitting position, at the L2-3 interspace, with an initial bolus of 20 mL of epidural 0.25% bupivacaine and continuous infusion of epidural 0.125% bupivacaine at a rate of 5 mL/h.

Anesthesia chart review revealed that, following an iv bolus of 2 mg of midazolam, TIVA was induced with a mean of 2.3 ± 0.1 mg/kg iv propofol and a mean of 2.2 ± 0.2 mcg/kg iv fentanyl. Neuromuscular block was induced with 0.6 mg/kg iv rocuronium and was maintained with regular bolus doses of 0.15 mg/kg iv every 30 minutes, except one patient, who required an additional dose every 15 minutes. According to anesthesia charts, anesthesia was maintained with 90–160 mcg/kg/min iv propofol infusion and 0.02–0.2 mcg/kg/min iv remifentanil infusion. Propofol infusion rate was adjusted to obtain a bispectral index value of 40–60. The mean bispectral index value obtained from the anesthesia charts was 43.9 ± 2.8. Remifentanil infusion rate was adjusted when blood pressure or heart rate varied.

### 3.2. Preoperative Hemodynamic Variables

Ward chart review for preoperative blood pressure and heart rate showed that 11 patients (4 in TIVA group and 7 in EA group) were hypertensive during the preoperative course, and 9 patients (4 in TIVA group, 5 in EA group) had a short course of supraventricular tachycardia, which required initiation of amiodarone therapy.

### 3.3. Preoperative Nutrition and Fluid Intake

Ward chart review for preoperative nutrition and fluid intake showed that 5 patients (15.6%), who had signs of pneumonia, received additional enteral nutrition (mean of 11.4 ± 1.2 kcal/kg/day). No patient received any form of parenteral nutrition. Urine output was not recorded during preoperative period.

### 3.4. Intraoperative Hemodynamic Variables

Intraoperative hemodynamic variables are summarized in Table 3. Anesthesia chart review showed that mean blood pressure dropped by a mean of 15.3 ± 15.3%. Drop in mean blood pressure was as high as 37–50% in 3 patients in TIVA group and in 2 patients in EA group. These patients required administration of iv ephedrine in addition to iv colloids. There was no occurrence of hypertensive, tachycardic, or bradycardic episode.

### 3.5. Intraoperative Fluid Intake and Urine Output

Regardless of the anesthesia method used, all patients received a median of 1125 ± 565 mL iv fluid during the operation according to the anesthesia chart (*P* = 0.9). Anesthesia charts showed that fluid loss during the preoperative fasting period was calculated according to patient's body weight. Patients received half of this volume during the first hour of surgery and the other half during the next two hours. Therefore, iv fluid administered during the operation was directly proportional to the duration of the operation, as analyzed by ANOVA (*P* < 0.0001). All patients received 500 mL of iv colloid, and the rest of iv fluids were isotonic sodium chloride 0.9%. Hourly urine output results during surgery showed that all patients had 1 mL/kg urine output following the first hour of surgery and there was no significant difference between groups (*P* = 0.8).

### 3.6. Postoperative Hemodynamic Variables

Postoperative hemodynamic variables are summarized in Table 4. Anesthesia charts and ICU records showed that patients in TIVA group continued to receive iv remifentanil infusion at a dose of 0.01 mcg/kg/min until their body temperature was 36.5°C (via active warming with heat blankets). During this period, which lasted for about 3 hours (160 ± 28 min), mean blood pressure and heart rate did not differ significantly between TIVA group (90 ± 15.3 mmHg, 81 ± 17 beats/min) and EA group (87 ± 14.2 mmHg, 76 ± 11 beats/min) (*P* = 0.4).

Charts showed that, after the warming period, mean blood pressure in TIVA group (103.5 ± 15.3 mmHg) significantly increased, when compared with EA group (85.9 ± 3.6 mmHg) (*P* = 0.0002). Also, mean heart rate in TIVA group (89.2 ± 17 beats/min) was significantly higher compared with EA group (76.4 ± 10.8 beats/min) (*P* = 0.018).

During the postoperative period, three patients in TIVA group (2 in the first day and 1 in the second day) had supraventricular tachycardia. These 3 patients were already receiving amiodarone treatment, and, according to charts, tachycardia was associated with pain, which was treated with rescue analgesics (tramadol 100 mg via iv infusion). There was no occurrence of hypotensive or bradycardic episode.

### 3.7. Postoperative Nutrition and Fluid Intake and Urine Output

The patients, who received less iv fluids due to short duration of surgery, received more fluids during the postoperative period in the ICU (*P* < 0.001). According to the ICU charts, all patients were ordered enteral feeding six hours after the end of the surgery. According to the charts, the standard meal consisted of low-fat, low-cholesterol, 2000 kcalories, and 60 grams of protein. The charts also showed that additional high energy oral nutritional supplements, four times a day (additional 1200 kcal/day), were ordered for nondiabetic patients, while diabetic patients were ordered additional diabetic supplements, four times a day (additional 960 kcal/day). Records showed that enteral feeding was not possible in 6 patients (33%) in TIVA group and one patient (7%) in EA group due to nausea, and vomiting was seen in 4 of these patients (all in TIVA group) despite treatment. Comparison of groups in terms of postoperative nausea and vomiting rate was statistically insignificant (*P* = 0.4). According to the charts, these 7 patients were treated with ondansetron and received infusion of 5% dextrose solution until the next day. During the ICU stay, mean iv fluid treatment (2193 ± 247 mL/day) and urine output (0.56 ± 0.08 mL/kg/h) were similar in both groups (*P* = 1 and 0.7, resp.).

### 3.8. Postoperative Analgesia and Numeric Rating Scores

According to patient records, TIVA group received 50 mg of iv tramadol, every six hours. EA group received continuous epidural infusion of 0.25% bupivacaine. Numeric rating scores were significantly high in TIVA group (5.4 ± 0.9, min: 4, max: 6) compared with EA group (1.8 ± 0.8, min: 0, max: 3) (*P* < 0.001). Also, 12 patients in TIVA group (70%) requested additional analgesics. Five of these patients received an iv infusion of 100 mg tramadol, and seven patients received intramuscular injection of 75 mg diclofenac sodium.

## 4. Discussion

This retrospective study showed that, compared to iv analgesia, epidural analgesia provides better pain control and enables earlier enteral feeding during postoperative period of endovascular repair of infrarenal abdominal aortic aneurysm.

### 4.1. Patient Demographics

Most patients were males. This is consistent with the current literature [6] since abdominal aortic aneurysm is more common in men of 65 years of age and older, and few women with an elective AAA are suitable for EVAR due to the anatomical differences [7].

Comorbidities were similar between groups, and hypertension, hyperlipidemia, and chronic obstructive pulmonary diseases were present in almost all patients. This is not surprising, since aneurysm is a vascular disease and smoking is strongly associated with formation and rupture of aortic aneurysm [1]. Although the chi-squared comparison of COPD presence between groups is not statistically significant, the abundance of COPD diagnosis in EA group is noticeable. Preanesthetic evaluation notes showed that all patients (except two) in the EA group had obstructive lung disease and were planned to be operated with regional anesthesia, because general anesthesia is more likely to cause atelectasis and postoperative respiratory failure [8]. One patient in the EA group had additional restrictive pathology (vital capacity: 600 mL, forced expiratory volume in the first second: 500 mL), needed continuous oxygen supply at a rate of 2 l/min to stay normoxemic, and therefore had an ASA score of IV. To prevent atelectasis, this patient received intermittent (6 times a day, for 30 min) continuous positive airway pressure support through a mask during surgery and the first two postoperative days in the ICU.

### 4.2. Anesthesia Method

The choice of TIVA instead of an inhaled anesthetic was obligatory for this hospital, since the gas scavenging system deployed for the anesthetic machine was insufficient. Midazolam and propofol are widely used in combination with fentanyl in angiographic interventions and EVAR [9]. However, the duration of surgeries in this hospital varied (85–230 min). Therefore, propofol and remifentanil were selected, because both of these drugs are easy to titrate and allow rapid arousal and extubation [10].

### 4.3. Intraoperative Hemodynamic Variables

The presence of hypertension despite regular use of antihypertensive treatment and tachycardic episodes in about one of three patients shows that some of these surgeries were semielective. A more detailed look at the demographic data revealed that the five patients, who had a drop in mean blood pressure more than 25% during the surgery, had both hypertension and coronary artery disease and had hypertensive and tachycardic episodes during the preoperative period. According to preoperative anesthesia note, three of these patients were operated with TIVA, because the surgeons expected a difficult and long surgery. Intraoperative blood pressure and heart rate in these patients varied more than their counterparts, which were operated with epidural anesthesia. Also, exactly these three patients required more iv fluids (>2 liters, although the duration of surgery was below the mean) and are responsible for the (albeit statistically insignificant) difference in the iv fluid requirement between groups.

### 4.4. Postoperative Hemodynamic Variables and Nutritional and Fluid Management

Mean blood pressure and heart rate were the same in both groups during immediate postoperative period, where the patients were warmed with heat blankets; the patients in EA group were treated with epidural infusion of local anesthetics, and the patients in TIVA group were still receiving analgesic dose of remifentanil.

However, after the cessation of remifentanil infusion, mean blood pressure and heart rate in TIVA group increased. It is understood that this hemodynamic response, which included tachyarrhythmias, was related to pain, since these patients were treated with analgesics instead of antihypertensive or antiarrhythmic drugs.

On the contrary, according to the charts, the patients in EA group were hemodynamically stable throughout the ICU stay. This stability was not associated with fluids or blood products, as both fluid therapy and urine output were indifferent between groups, and none of the patients required postoperative transfusion. In our opinion, the hemodynamic stability was the result of improved analgesia. This was evident because none of the patients in EA group requested rescue analgesics, and the numeric rating scores were lower in EA group compared to TIVA group.

Only one patient in EA group experienced postoperative nausea and vomiting, whereas one-third of patients in TIVA group could not be fed enterally during the first day due to nausea and vomiting. The high occurrence of nausea may be attributed to tramadol, which is reported to cause dose-dependent nausea and vomiting, especially during the initial treatment [11]. This view may be supported by the fact that nausea was dominant after the warming period, where the patients in TIVA group continued to receive remifentanil infusion. Nausea may also have occurred due to inadequate analgesia, as there were recordings of nausea in 3 patients in TIVA group, who were treated with rescue infusion of tramadol due to tachycardia. Since these 3 patients were treated with additional tramadol dose, the mechanism of nausea in these patients may not be clear. Nausea may have occurred either due to inadequate analgesia or due to tramadol or due to the hemodynamic instability. In our opinion, epidural analgesia did not cause as much nausea as iv analgesia (although statistically not significant), and hemodynamic stability and earlier feeding may be the main reasons of the significantly shorter stay in the ICU.

## 5. Conclusions

In conclusion, this retrospective case-control study found that epidural anesthesia and postoperative epidural analgesia better reduce postoperative pain compared with total iv anesthesia and systemic analgesia, with similar effects on hemodynamic status.

## Figures and Tables

**Table 1:** Numeric rating scale.

Rating	Pain level
0	No pain
1–3	Mild pain (nagging, annoying, and interfering a little with daily activities)
4–6	Moderate pain (interferes significantly with daily activities)
7–10	Severe pain (disabling)

**Table 2:** Patient demographics obtained from patient charts. Data are represented as mean ± SD, number, or number (percent).

	TIVA	Epidural anesthesia	*P* value
	(*n* = 17)	(*n* = 14)	
Age	71 ± 7.5	75.8 ± 6.4	NA
Gender (male/female)	15/2	12/2	NA
ASA score (II/III/IV)	1/15/0	6/8/1	NA
Comorbidities			
Hypertension	15 (88%)	13 (93%)	NA
Hyperlipidemia	15 (88%)	13 (93%)	NA
Coronary artery disease	13 (76%)	3 (21%)	NA
Diabetes mellitus	4 (23%)	3 (21%)	NA
Chronic obstructive pulmonary disease	9 (53%)	12 (86%)	

**Table 3:** Duration of operation, hemodynamic variables, and complications recorded during the operations. Data are represented as median ± SD or number (percent).

	TIVA	Epidural anesthesia	*P* value
	(*n* = 17)	(*n* = 14)	
Mean duration of operation (min)	140 ± 44	125 ± 31	NA
Mean blood pressure (mmHg)	83 ± 13	80 ± 15	NA
Mean heart rate (beats/min)	80 ± 14	80 ± 10	NA
Median intravenous fluid (mL)	1250 ± 615	875 ± 485	NA
Mean urine output (mL)	350 ± 50	400 ± 60	NA
Median red blood cells transfused (units)	0 ± 2	0 ± 0	NA
Number of hypotensive episodes	3 (17%)	2 (14%)	NA

**Table 4:** Total postoperative analgesic drug, hemodynamic variables, and complications recorded during the postoperative intensive care unit (ICU) stay. Data are represented as median ± SD, mean ± SD, or number (percent).

	TIVA	Epidural anesthesia	*P* value
	(*n* = 17)	(*n* = 14)	
Mean duration of ICU stay (days)	5 ± 2	3 ± 1	NA
Total analgesic used	8.6 ± 1.7 mg remifentanil	54.5 ± 12.3 mg bupivacaine	NA
Mean blood pressure (mmHg)	103.5 ± 15.3	85.9 ± 3.6	0.0002
Mean heart rate (beats/min)	89.2 ± 17	76.4 ± 10.8	0.018
Median red blood cells transfused (units)	None	None	NA
Occurrence of nausea and vomiting	6 (33%)	1 (7%)	0.15
